# Association between phosphorus intake and wrist fracture: the mediating role of bone mineral density

**DOI:** 10.29219/fnr.v70.13272

**Published:** 2026-07-31

**Authors:** Xiaohui Niu, Rongcheng Xu, Xuanyu Mao, Guangyi Zou

**Affiliations:** Department of Trauma Orthopedics, Lishui Hospital of Wenzhou Medical University, The First Affiliated Hospital of Lishui University, Lishui People’s Hospital, Lishui, Zhejiang 323000, China

**Keywords:** phosphorus, wrist fracture, bone mineral density, mediation analysis, NHANES

## Abstract

**Background:**

Phosphorus correlates with bone mineral density (BMD), a key factor in fracture risk. Thus, BMD may mediate phosphorus–fractures association, which has not yet been elucidated.

**Objective:**

This study aimed to trace phosphorus intake–wrist fracture association, and analyze the mediating role of BMD.

**Methods:**

Data were derived from the National Health and Nutrition Examination Survey (NHANES) database. Logistic regression and restricted cubic spline (RCS) analyses were utilized to assess phosphorus intake–wrist fracture association. Subgroup analyses examined differences across populations with varying characteristics. Mediation analysis explored the potential mediating role of BMD in the phosphorus–wrist fracture association.

**Results:**

A total of 7,952 participants were included. In the fully adjusted model, compared with the Q1 group, the Q4 group had a remarkably reduced wrist fracture risk (odds ratio [OR] = 0.58, 95% confidence interval [CI]: 0.38–0.88, *P* = 0.011). RCS revealed a non-linear association between phosphorus intake and wrist fracture (*P*-non-linear = 0.026). When phosphorus intake was <1,086.5 mg/day, higher intake correlated with lower wrist fracture risk; no significant negative association was observed beyond the threshold. In addition, no subgroups with interactions were identified (*P*-interaction > 0.05). Mediation analysis indicated that BMD mediated 12.1% of the association between phosphorus intake and wrist fracture.

**Conclusion:**

When phosphorus intake is <1,086.5 mg/day, higher intake is associated with reduced wrist fracture risk, with BMD as a key mediator. These findings provided crucial scientific evidence for developing targeted nutritional strategies to prevent wrist fractures.

## Popular scientific summary

- This study is the first to reveal a nonlinear threshold association between dietary phosphorus intake and wrist fracture risk. This finding provides new evidence for understanding the complex relationship between phosphorus and fractures and suggests that optimal phosphorus intake should be considered when formulating dietary recommendations or clinical interventions, especially in elderly and female populations, and may help reduce wrist fracture risk by maintaining bone density.

Wrist fracture, defined as a break or fissure in any bone constituting the wrist joint, is a common upper-limb fracture (1, 2). According to the 2017 Global Burden of Disease Study, the age-standardized incidence of hand and wrist fractures is 179 per 100,000 persons (3). Wrist fracture risk escalates with age. In the U.S., its prevalence reaches 12% among adults aged ≥50 years, and 17.8% of individuals experience a second wrist fracture after the first (4). More critically, persistent pain, functional impairment, and increased healthcare utilization lasting ≥12 months severely compromise life quality for many patients (5). Thus, identifying modifiable risk factors is urgent for developing preventive strategies.

Dietary factors, due to their modifiability, quantifiability, and lifelong relevance, have been extensively studied in bone health maintenance (6). Phosphorus, the second most abundant mineral in humans (≈1% of total body weight), is primarily deposited (≈85%) as hydroxyapatite in bones and teeth, providing rigidity and compressive strength to the bone matrix (7, 8). Previous studies suggest a close association between phosphorus and fracture risk. Subjects with serum phosphorus levels in the low-normal range or significantly below normal exhibit increased fracture risk compared to those with high-normal levels (9). Conversely, a study reports that higher serum phosphorus levels correlate with elevated fracture risk (10). Given these conflicting results, further exploration of the specific relationship between phosphorus intake and wrist fracture is essential. Notably, phosphorus intake modulates bone mineral density (BMD) in the host (11), a key pathological factor in fracture susceptibility (12). We thus hypothesize that BMD likely mediates the link between phosphorus exposure and wrist fracture. However, the dose-response relationship between phosphorus exposure and wrist fracture is currently unclear, and there is a lack of exploration into potential non-linear associations. In addition, the role of BMD as a key mediating pathway has not been studied and quantified in population-based studies.

Therefore, leveraging the National Health and Nutrition Examination Survey (NHANES) database, we investigated the dose-response relationship of phosphorus intake and wrist fracture and further analyzed non-linear features and specific thresholds. In addition, this study is the first to examine and quantify the mediating role of BMD in the association between the two in epidemiological research, aiming to provide new evidence-based support for precise nutritional prevention of wrist fractures.

## Methods

### Study population

Data were sourced from NHANES, a cross-sectional, stratified, multistage probability sampling survey designed to systematically assess the health and nutritional status of the non-institutionalized U.S. population. All study protocols were approved by the National Center for Health Statistics Ethics Review Board with written informed consent from all participants. Thus, additional ethical approval was unnecessary.

**Fig. 1:**
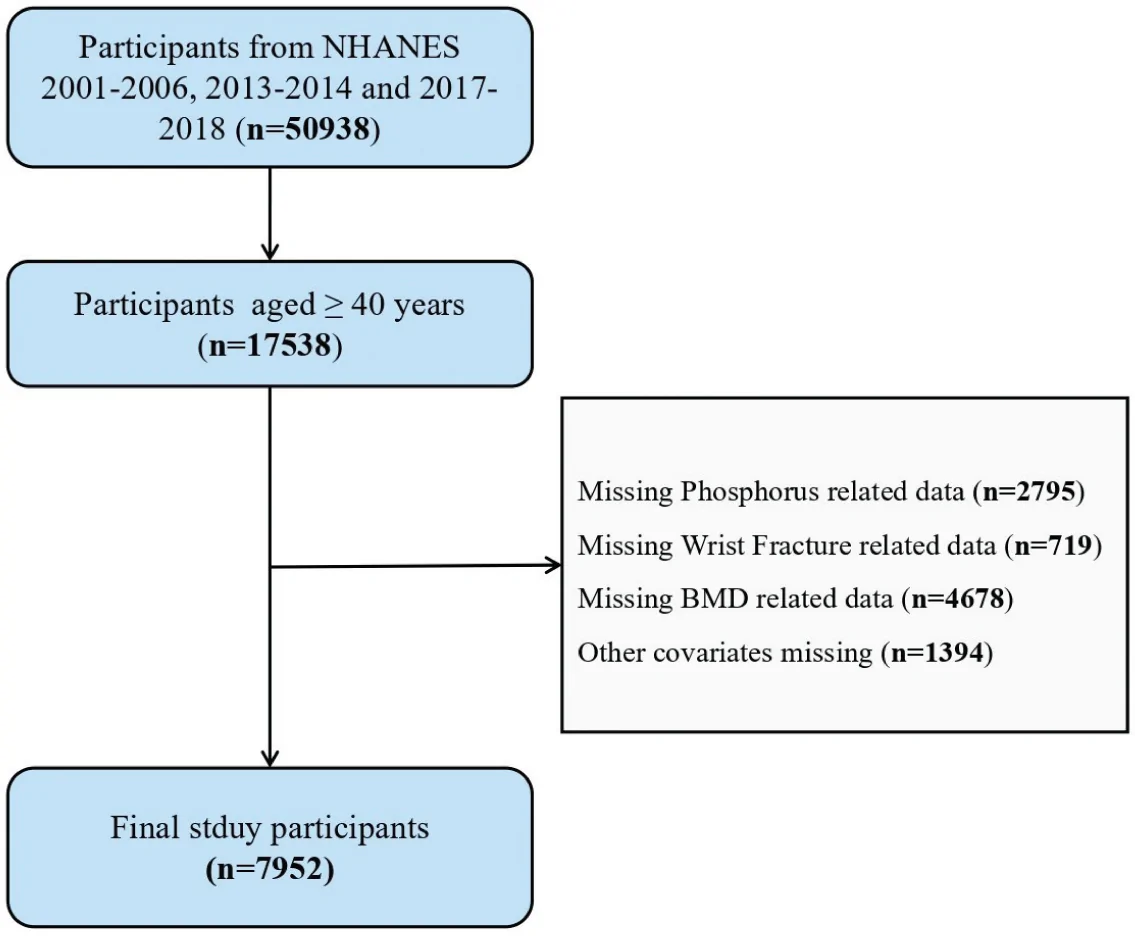
Flowchart of participant inclusion and exclusion.

Data from the survey cycles (2001–2006, 2013–2014, and 2017–2018), including all major variables needed by this study, were used to explore the relationship between phosphorus intake and wrist fracture (*n* = 50,938). After excluding individuals aged <40 years (*n* = 33,400) and those with missing data on phosphorus (*n* = 2,795), wrist fracture (*n* = 719), BMD (*n* = 4,678), and other covariates (*n* = 1,394), 7,952 participants were included (Fig. 1).

### Phosphorus intake

Phosphorus intake data were collected via 24-h dietary recalls (13). Each participant underwent two interviews: an initial face-to-face interview and a telephone follow-up 3–10 days later. The average phosphorus intake from both recalls was used for analysis. If data from the second interview were missing, data from the first were used. Outliers greater than or less than the weighted mean ± 3 standard deviations were treated as missing.

Phosphorus intake was categorized into gender-specific quartiles. Females: Q1: <816.5, Q2: 816.5–1,074, Q3: 1,074–1,363.5, Q4: ≥1,363.5; Males: Q1: <1,092, Q2: 1,092–1,420.5, Q3: 1,420.5–1,777, Q4: ≥1,777(unit: mg/day).

### Wrist fracture

Participants were asked if they had ever been diagnosed with a wrist fracture by an orthopedic specialist. Those answering ‘yes’ were classified as having wrist fracture (14).

### BMD

All participants underwent BMD testing via dual-energy X-ray absorptiometry. Data were analyzed using Hologic APEX software (v4.0) (15). Detailed protocols are available on the NHANES website (https://wwwn.cdc.gov/Nchs/Data/Nhanes/Public/2013/DataFiles/DXX_H.htm).

### Covariates

Covariates comprised demographic characteristics, life styles, laboratory indicators, and comorbidities (Table 1).

**Table 1:** Baseline characteristics of participants

Characteristics	Overall	Q1	Q2	Q3	Q4	*P* (value)
*N*	7,952	2,331	2,076	1,789	1,756	
Age (Mean, SD) (year)	54.6 (10.4)	56.8 (11.2)	55.4 (10.8)	54.0 (9.8)	52.3 (9.2)	<0.001
**Gender (%)**						0.999
Female	4,050 (52.3)	1,147 (52.2)	1,083 (52.4)	920 (52.3)	900 (52.4)	
Male	3,902 (47.7)	1,184 (47.8)	993 (47.6)	869 (47.7)	856 (47.6)	
**Race (%)**						<0.001
Mexican American	1,411 (5.6)	387 (5.3)	385 (5.9)	292 (5.1)	347 (6.0)	
Non-Hispanic White	4,228 (75.5)	1,105 (69.5)	1,097 (75.4)	1,001 (77.1)	1,025 (80.0)	
Non-Hispanic Black	1,481 (9.8)	608 (15.2)	370 (9.7)	292 (8.5)	211 (6.0)	
Other Race	832 (9.1)	231 (10.0)	224 (9.1)	204 (9.3)	173 (8.0)	
**Education (%)**						<0.001
Middle school and below	1,016 (5.5)	412 (8.8)	278 (6.0)	162 (3.8)	164 (3.5)	
High school	1,032 (9.7)	387 (13.2)	252 (9.6)	197 (8.4)	196 (7.6)	
College and above	5,904 (84.8)	1,532 (78.0)	1,546 (84.5)	1,430 (87.8)	1,396 (88.9)	
**Marital status (%)**						0.014
Single	2,662 (29.7)	860 (33.4)	704 (28.7)	543 (28.2)	555 (28.2)	
Coupled	5,290 (70.3)	1,471 (66.6)	1,372 (71.3)	1,246 (71.8)	1,201 (71.8)	
PIR (Mean, SD)	3.3 (1.6)	3.0 (1.6)	3.3 (1.6)	3.5 (1.5)	3.5 (1.5)	<0.001
**Smoke (%)**						<0.001
Non-smoker	3,885 (49.6)	1,064 (45.1)	1,004 (50.0)	904 (49.6)	913 (53.7)	
Former smoker	2,437 (29.8)	723 (29.7)	646 (29.6)	542 (29.0)	526 (31.1)	
Current smoker	1,630 (20.5)	544 (25.2)	426 (20.3)	343 (21.4)	317 (15.2)	
**Alcohol (%)**						<0.001
Non-drinker	2,940 (30.7)	1,048 (38.8)	761 (31.3)	562 (25.2)	569 (27.4)	
Current drinker	5,012 (69.3)	1,283 (61.2)	1,315 (68.7)	1,227 (74.8)	1,187 (72.6)	
BMI (Mean, SD) (kg/m^2^)	28.9 (6.3)	28.9 (6.3)	28.8 (6.1)	28.6 (6.0)	29.4 (6.9)	0.109
**Hypertension (%)**						<0.001
No	3,851 (52.5)	978 (45.9)	979 (51.9)	929 (54.5)	965 (57.7)	
Yes	4,101 (47.5)	1,353 (54.1)	1,097 (48.1)	860 (45.5)	791 (42.3)	
**Diabetes (%)**						0.006
No	6,517 (86.7)	1,860 (84.0)	1,686 (85.9)	1,522 (88.8)	1,449 (88.2)	
Yes	1,435 (13.3)	471 (16.0)	390 (14.1)	267 (11.2)	307 (11.8)	
25(OH) D (Mean, SD) (nmol/L)	64.4 (23.8)	58.9 (23.3)	63.9 (23.2)	66.3 (22.9)	68.7 (24.5)	<0.001
Total calcium (Mean, SD) (mmol/L)	2.37 (0.09)	2.37 (0.10)	2.37 (0.10)	2.37 (0.09)	2.37 (0.09)	0.896
Total energy intake (Mean, SD) (kcal)	2033.8 (772.3)	1367.3 (459.3)	1837.8 (482.3)	2188.3 (591.8)	2744.0 (763.0)	<0.001
BMD (Mean, SD) (g/cm^2^)	1.13 (0.12)	1.11 (0.13)	1.12 (0.12)	1.13 (0.12)	1.15 (0.12)	<0.001

Female: Q1: < 816.5, Q2: 816.5 ~ 1,074, Q3: 1,074 ~ 1363.5, Q4: ≥ 1363.5.

Male: Q1: < 1,092, Q2: 1,092 ~ 1420.5, Q3: 1420.5 ~ 1,777, Q4: ≥ 1,777.

### Statistical analysis

All statistical analyses followed NHANES guidelines for analyzing and reporting complex survey data, with the *survey* Rvpackage (version 4.1.3) used to calibrate complex sampling designs (including stratification, clustering, and weighting). Since the main exposure variable in this study was phosphorus intake, we used 2-day dietary recall weights. To merge multiple survey cycles, we created new analysis weights based on NHANES guidelines. Specifically, the weight of the original 2-day dietary recall (WTDRD1 for the 2001–2002 cycle and WTDR2D for the other cycles) divided by the number of merged cycles [3], yielding the new weight=WTDR2D/3. This operation ensured that each cycle had proportional representativeness in the merged sample. In the analysis, we specified stratified variables (SDMVSTRA), primary sampling unit variables (SDMVPSU), and the newly created dietary weights mentioned above. Dietary weights were applied. Weighted baseline characteristics were tabulated using the ‘tableone’ package. Categorical variables were presented as *N* (weighted %), continuous variables as weighted mean (SD), compared across groups employing chi-square tests and ANOVA. Logistic regression was utilized to evaluate the effect of phosphorus intake on wrist fracture. Non-linear associations were assessed utilizing the ‘rms’ package to implement RCS, with threshold analysis testing for segmented relationships. By using the ‘jstable’ package, subgroup analyses were performed to examine age and gender effects. We conducted mediation analysis by the ‘mediation’ package to explore the possible mediating role of BMD.

Statistical significance defined as *P* < 0.05.

## Results

### Baseline characteristics

Among 7,952 participants, there was a roughly equal ratio of males to females, and mean age was 54.6 ± 10.4 years (Table 1). Compared to the lowest phosphorus intake group (Q1), Q2–Q4 groups had higher BMD (*P* < 0.001). Additionally, Q2–Q4 groups were younger and had higher proportions of advanced education, marriage, non-smoking, alcohol consumption, absence of hypertension or diabetes, higher poverty-income ratios, higher 25-hydroxyvitamin D levels, and greater total energy intake (Table 1).

### Association between phosphorus intake and wrist fracture

Table 2 presents the association. In the fully adjusted Model 3, compared with Q1, wrist fracture risk in higher intake groups Q2, Q3, and Q4 were reduced by 25% (OR = 0.75, 95% CI: 0.58–0.99, *P* = 0.041), 45% (OR = 0.55, 95% CI: 0.38–0.81, *P* = 0.003), and 42% (OR = 0.58, 95% CI: 0.38–0.88, *P* = 0.011), respectively.

**Table 2:** Association between phosphorus intake and wrist fracture

Phosphorus	Model 1 OR (95% CI)	*P* (value)	Model 2 OR (95% CI)	Model 3		
				*P* (value)	OR (95% CI)	*P* (value)
Q1	Ref		Ref		Ref	
Q2	0.90 (0.68~1.19)	0.436	0.86 (0.65~1.13)	0.273	**0.75 (0.58~0.99)**	**0.041**
Q3	0.75 (0.55~1.02)	0.068	**0.70 (0.50~0.97)**	**0.031**	**0.55 (0.38~0.81)**	**0.003**
Q4	0.93 (0.71~1.23)	0.614	0.84 (0.62~1.15)	0.272	**0.58 (0.38~0.88)**	**0.011**

Female: Q1: < 816.5, Q2: 816.5 ~ 1,074, Q3: 1,074 ~ 1363.5, Q4: ≥ 1363.5.

Male: Q1: < 1,092, Q2: 1,092 ~ 1420.5, Q3: 1420.5 ~ 1777, Q4: ≥ 1,777.

Model 1: Unadjusted; Model 2: Adjusted for age, gender, race, education, marital status, PIR; Model 3: Adjusted smoke, alcohol, BMI, hypertension, diabetes, 25(OH)D, total calcium, total energy intake based on Model 2. Statistically significant results are highlighted in bold.

**Fig. 2:**
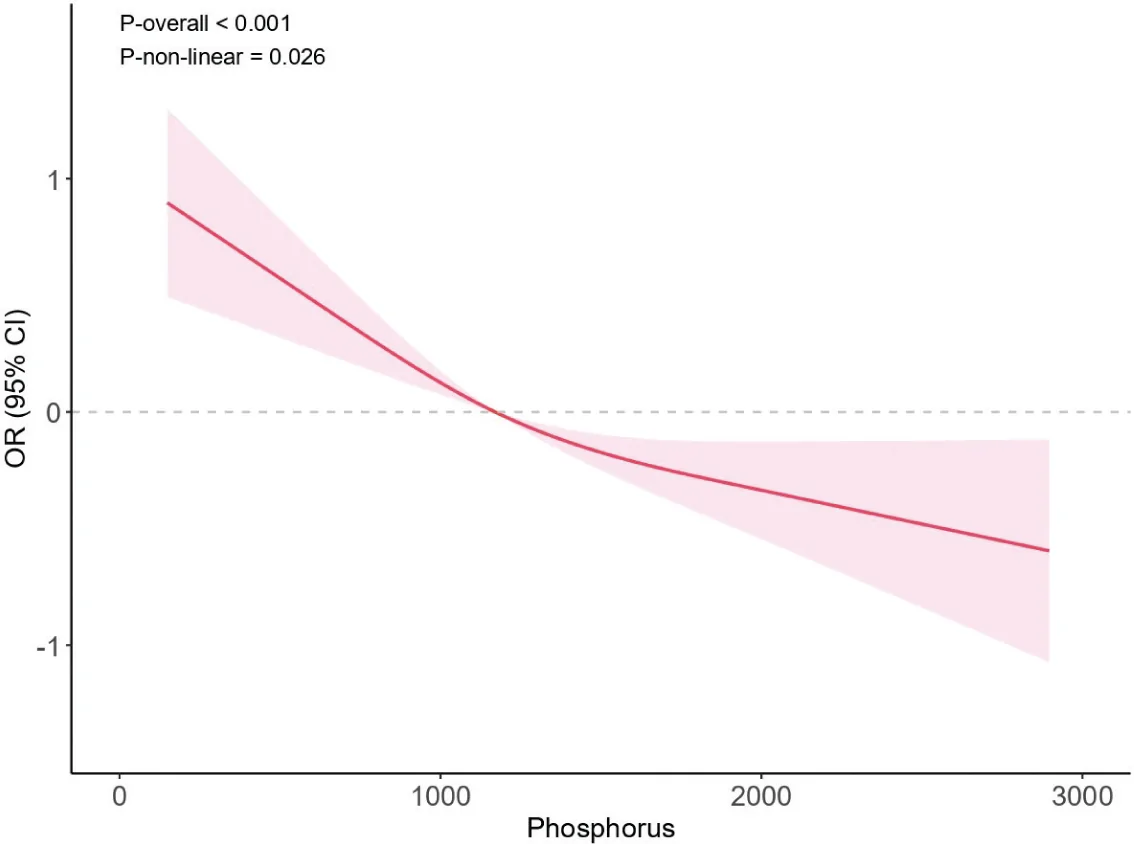
RCS curve of phosphorus intake and wrist fracture risk.

RCS showed a marked overall association (*P*-overall < 0.001) with significant non-linearity (*P*-non-linear = 0.026) (Fig. 2). We identified an inflection point at 1,086.5 mg/day using threshold analysis. Below this point, higher phosphorus intake correlated with progressively lower wrist fracture risk (OR = 0.999, 95% CI: 0.998–0.999, *P* < 0.001). Above it, no significant negative association existed (Table 3).

**Table 3:** Threshold effect analysis of phosphorus intake and wrist fracture

Phosphorus	Adjusted OR (95% CI)	*P* (value)
Model 1	1.000 (0.999~1.000)	<0.001
Model 2		
Inflection point (1086.5)		
<1086.5	0.999 (0.998~0.999)	<0.001
>1086.5	1.000 (0.999~1.000)	0.017
P (LR)		<0.001

Model 1: Fitting model by standard linear regression.

Model 2: Fitting model by two-piecewise linear regression.

P (LR): P for likelihood ratio test.

### Subgroup analysis

No subgroups showed notable interaction (*P*-interaction > 0.05) (Table 4). RCS indicated noticeable overall associations in all subgroups. Non-linear associations were found in participants aged ≥ 60 years (*P*-non-linear = 0.003) and females (*P*-non-linear = 0.002) (Supplementary Fig. 1). Threshold points were 1,616.0 mg/day (age ≥ 60) and 1,150.5 mg/day (females). Below thresholds, higher intake reduced risk; above thresholds, negative associations became non-significant (Supplementary Table 1).

**Table 4:** Subgroup analysis stratified by age and gender

Subgroup	Count	Level	OR (95% CI)	*P* (value)	*P* (interaction)
**Age**					0.967
<60 years	4,964	Q1	Ref		
		Q2	0.71 (0.51~0.99)	0.046	
		Q3	0.62 (0.39~0.96)	0.038	
		Q4	0.63 (0.39~1.02)	0.068	
≥60 years	2,988	Q1	Ref		
		Q2	0.84 (0.56~1.25)	0.395	
		Q3	0.37 (0.21~0.66)	0.002	
		Q4	0.43 (0.26~0.72)	0.004	
**Gender**					0.867
Female	4,050	Q1	Ref		
		Q2	0.64 (0.44~0.93)	0.023	
		Q3	0.50 (0.31~0.79)	0.004	
		Q4	0.60 (0.32~1.12)	0.115	
Male	3,902	Q1	Ref		
		Q2	0.88 (0.61~1.28)	0.506	
		Q3	0.60 (0.37~0.97)	0.041	
		Q4	0.57 (0.34~0.94)	0.034	

### Mediation analysis

**Fig. 3:**
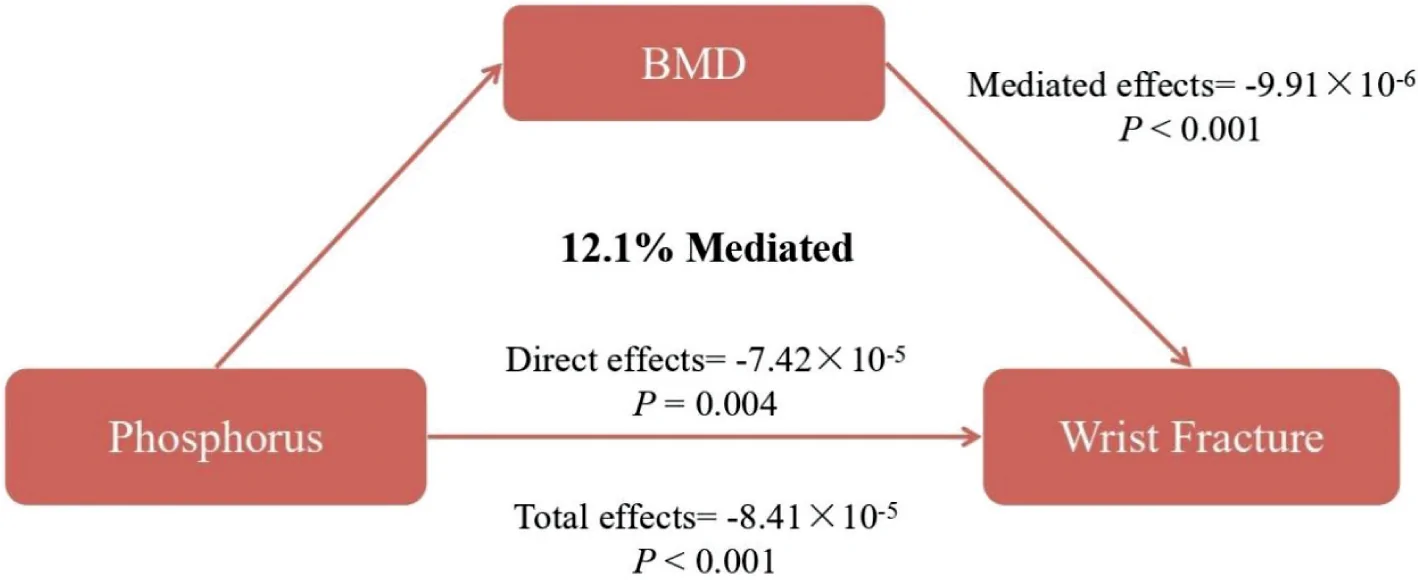
Mediating role of BMD in the association between phosphorus intake and wrist fracture.

Mediation analysis demonstrated that BMD mediated the association between phosphorus intake and wrist fracture, with a mediation proportion of 12.1% (Fig. 3).

## Discussion

Based on nationally representative, large-sample data, this study examined the relationship between dietary phosphorus intake and wrist fracture risk and revealed a non-linear association with an inflection point at 1,086.5 mg/day. Specifically, wrist fracture risk decreased as phosphorus intake rose to this level, whereas no further reduction was seen beyond it. This pattern persisted among participants aged ≥ 60 years and among women. BMD mediated 12.1% of the phosphorus–fracture association.

This significant non-linear threshold provides new evidence that reconciles earlier contradictory findings. There are various opinions regarding the association between dietary phosphorus and bone health. Acute adverse effects of high phosphorus on calcium and bone metabolism have been reported, with overall fracture risk increased for every 100 mg increase of intake (OR 1.09) (16). However, low phosphorus intake has also been revealed to correlate with microstructural deterioration, such as thinning of bone trabeculae (17). Inconsistent long-term associations are emphasized in clinic data (18, 19). A U-shaped relation of phosphorus intake and bone fracture has been suggested, with one analysis setting the threshold for risk changes at 939.44 mg/day (20). However, direct evidence for wrist fracture and phosphorus intake was lacking.

We proposed that the high heterogeneity of previous research is likely due to systematic differences in the distribution of baseline phosphorus intake levels among different study populations, which was reflected by different segments of the dose-response curve (inflection point of 1,086.5 mg/day) that we revealed. Specifically, when the dietary phosphorus intake of a cohort is generally below this threshold, researchers are more likely to observe a significant negative correlation between phosphorus intake and the risk of wrist fractures, which is consistent with the biological mechanism of correcting phosphorus deficiency to improve bone mineralization (21, 22). On the contrary, if the intake level of the study population generally exceeds or crosses this threshold, the potential disruption of calcium-phosphorus balance and negative effects on bone metabolism caused by high phosphorus intake (19, 23) will weaken or even offset the protective effect, resulting in no significant correlation or a weak positive correlation trend. Therefore, our non-linear model and identified specific thresholds integrate previous findings into a unified framework with an optimal intake window, providing critical dose-response evidence for conflicting conclusions.

Biologically, the non-linearity is plausible. Inadequate intake can precipitate hypophosphatemia, impair mineralization, induce osteomalacia, and compromise chondrocyte differentiation and callus formation during fracture repair, thereby increasing wrist fracture risk (17, 24). Therefore, increasing phosphorus intake at this stage is crucial for bone health. However, excessive phosphorus intake may weaken its protective effect through multiple pathways. For example, hyperphosphatemia can inhibit the synthesis of active vitamin D and reduce intestinal absorption of calcium (25), while reduced calcium absorption can exacerbate the risk of fractures (26). Excessive phosphorus intake may also stimulate excessive secretion of parathyroid hormone, leading to secondary hyperparathyroidism, promoting increased osteoclast activity, causing bone destruction and loss of bone mass (19, 23). Therefore, there is indeed an optimal phosphorus intake window for preventing wrist fractures. More intake does not simply indicate better effects.

The non-linear association persisted in individuals aged ≥ 60 years and females, with protective effects diminishing beyond thresholds (1,616.0 mg/day and 1,150.5 mg/day, respectively). Aging reduces glomerular filtration rate, impairing renal function (27). Excess phosphorus intake can induce or exacerbate chronic kidney disease, disrupting calcium-phosphorus metabolism and causing bone mineralization, linear growth, or abnormal strength, thereby increasing wrist fracture risk (28, 29). Concurrently, reduced bone formation capacity, severe bone loss, and impaired bone quality occur in the aged (30). Under these conditions, excess phosphorus further suppresses osteoblast activity and promotes osteoclast differentiation, heightening wrist fracture risk. Females typically have higher serum phosphorus levels than males (31), especially postmenopausal women experiencing rapid increases (32). Thus, excessive intake more readily causes phosphorus-calcium imbalance and hyperphosphatemia, increasing wrist fracture risk. Females also generally consume less calcium than males (33). Low calcium intake (<400 mg/day) increases sensitivity to phosphorus, while every 100 mg phosphorus increase raises fracture risk by 9% (16), amplifying phosphorus’s impact on wrist fracture risk. Therefore, older adults and females should strictly control phosphorus intake to avoid excess. (34)

BMD mediates the phosphorus–wrist fracture association. A cross-sectional study shows phosphorus intake positively correlates with bone mineral content and BMD, reducing osteoporosis risk in individuals >20 years (34). An animal study indicates that an increased phosphorus intake elevates BMD at 3 weeks and enhances bone structural strength at 8 weeks, maximizing fracture resistance (35). Although the exact mechanisms remain incompletely elucidated, several factors are key. As noted, phosphorus forms calcified phosphate minerals in bone tissue, a primary inorganic component, increasing BMD and strength, thus mitigating wrist fracture risk. Phosphorus also promotes protein synthesis and cellular metabolism, accelerating bone tissue repair and regeneration (36), further increasing BMD and reducing risk. Additionally, phosphorus maintains bone metabolic balance by regulating parathyroid hormone, active vitamin D, and fibroblast growth factor 23 (7), influencing BMD and providing protection against wrist fracture. However, specific signaling pathways require further validation.

In summary, the main contribution of this study is to break through the limitations of traditional linear models by identifying specific dose-response thresholds (1,086.5 mg/day) in the association between dietary phosphorus and wrist fractures, and confirming that BMD plays a partial mediating role (12.1%), thus establishing a more accurate and mechanistic association model. These results suggest that when developing dietary guidelines related to bone health, attention should be paid to the appropriate range of nutrient intake. The study also provides important empirical evidence for developing differentiated prevention strategies for populations with different levels of phosphorus intake. Nevertheless, limitations of this study persist. Firstly, the cross-sectional design captured the static co-occurrence relationship between phosphorus intake and wrist fracture, but it cannot dynamically track temporal evolution or establish reliable causal inference. Prospective cohorts are needed to validate causality. Secondly, NHANES estimated daily phosphorus intake via two non-consecutive 24-h recalls, which, while widely used in large epidemiological surveys, may reduce recall bias. Thirdly, despite extensive covariate adjustment, unrecorded factors influencing BMD (e.g. genetic background, medication history) may cause residual confounding. Finally, although we identified the mediating role of BMD, the precise mechanisms remain unclear. Multi-omics data could be leveraged in deeper analysis.

## Conclusion

Phosphorus intake below 1,086.5 mg/day is associated with substantially reduced wrist fracture risk as intake increases, with BMD playing a mediating role. These findings provide novel evidence-based insights for developing rational phosphorus interventions to optimize BMD and reduce wrist fracture risk.

## Supplementary Material



## Declaration

### Data availability statement

The data that support the findings of this study are available from the corresponding author upon reasonable request.
