# Supplementary material for: Association between phosphorus intake and wrist fracture: the mediating role of bone mineral density
Source: Food Nutr Res. 2026 Jul 31;70:10.29219/fnr.v70.13272. doi: 10.29219/fnr.v70.13272 (PMC13563442; doi:10.29219/fnr.v70.13272)
Supplement: Supplementary file 1 [file FNR-70-13272-s1.zip › 13272-Supplementary Material.pdf]

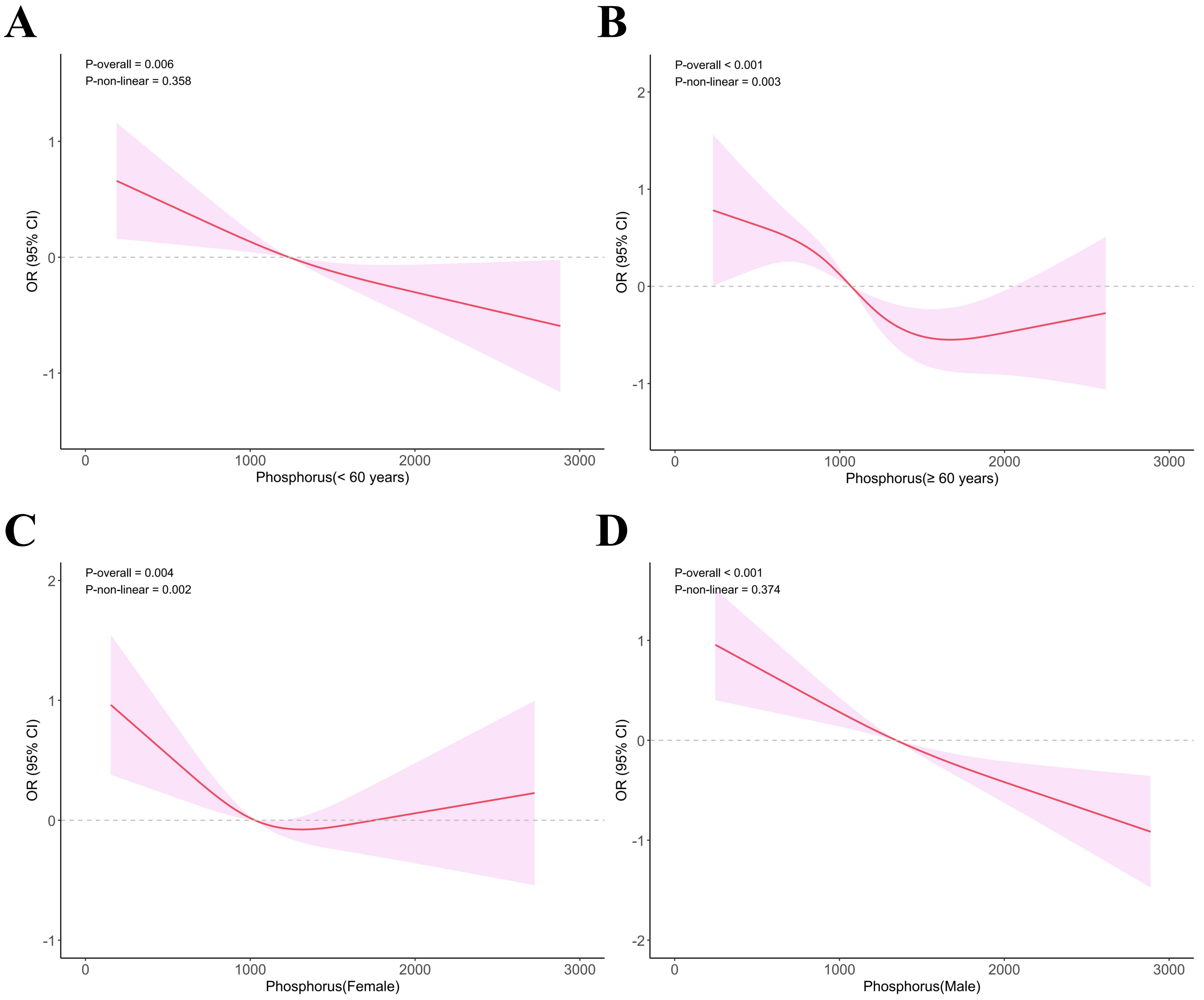
A
P-overall = 0.006
P-non-linear = 0.358
OR (95% CI)
Phosphorus(< 60 years)
B
P-overall < 0.001
P-non-linear = 0.003
OR (95% CI)
Phosphorus(≥ 60 years)
C
P-overall = 0.004
P-non-linear = 0.002
OR (95% CI)
Phosphorus(Female)
D
P-overall < 0.001
P-non-linear = 0.374
OR (95% CI)
Phosphorus(Male)
