# Supplementary material for: Association between phosphorus intake and wrist fracture: the mediating role of bone mineral density
Source: Food Nutr Res. 2026 Jul 31;70:10.29219/fnr.v70.13272. doi: 10.29219/fnr.v70.13272 (PMC13563442; doi:10.29219/fnr.v70.13272)
Supplement: Supplementary file 1 [file FNR-70-13272-s1.zip › 13272-Supplementary Material-92151-1-11-20251019.docx]

**Table S1 Table S1 Threshold Effect Analysis of Phosphorus Intake and Wrist Fractures in Aged ≥60 years and Female Population**

| Phosphorus | Adjusted OR (95% CI) | P (value) |
| --- | --- | --- |
| **≥ 60 years** |  |  |
| Model 1 | 0.999 (0.999~1.000) | 0.003 |
| Model 2 |  |  |
| Inflection point (1616) |  |  |
| <1616 | 0.999 (0.998~0.999) | <0.001 |
| >1616 | 1.000 (1.000~1.001) | 0.261 |
| P(LR) |  | <0.001 |
|  |  |  |
| **Female** |  |  |
| Model 1 | 1.000 (0.999~1.000) | 0.192 |
| Model 2 |  |  |
| Inflection point (1150.5) |  |  |
| <1150.5 | 0.999 (0.998~0.999) | 0.001 |
| >1150.5 | 1.000 (1.000~1.001) | 0.299 |
| P (LR) |  | <0.001 |
